# Smooth Muscle Tumor Originating in the Pleura: A Case Report and Updated Literature Review

**DOI:** 10.1155/2016/9581395

**Published:** 2016-09-22

**Authors:** Santiago Fabián Moscoso Martínez, Vadim Zarubin, Geethapriya Rajasekaran Rathnakumar, Alireza Zarineh

**Affiliations:** ^1^Department of Hematology and Oncology, The Brooklyn Hospital Center, 121 Dekalb Ave, New York, NY 11201, USA; ^2^Department of Internal Medicine, The Brooklyn Hospital Center, 121 Dekalb Ave, New York, NY 11201, USA; ^3^Department of Pathology, The Brooklyn Hospital Center, 121 Dekalb Ave, New York, NY 11201, USA

## Abstract

Smooth muscle tumors (SMTs) of the pleura are exceptionally rare. At present and to the best of these authors' knowledge, there are only 17 cases reported in the literature. We describe a case of a 51-year-old woman who complained of left sided pleuritic chest pain. Further, computed tomography (CT) revealed a left sided localized pleural-based mass involving the 9th rib. She underwent an interventional radiology guided percutaneous core biopsy of the lesion, which disclosed a “Smooth Muscle Tumor of Undetermined Malignant Potential (SMT-UMP).” A video-assisted thoracoscopic surgery (VATS) was performed for diagnosis and treatment purposes. Resections of the pleural-based mass and 9th rib were performed. SMT-UMP was the definitive diagnosis.

## 1. Introduction

Intrathoracic smooth muscle tumors are uncommon in the respiratory tract (upper and lower respiratory tract). They are seen occasionally in the gastrointestinal tract and commonly seen in the urogenital system [1–3]. However, the existence and diagnostic criteria of smooth muscle tumors originating in the pleura have been controversial and only rare and sporadic case reports have been mentioned in the literature [4].  Table 1 describes all cases that have been reported in the literature. We present a case of SMT-UMP with CT evidence of involvement of the 9th rib.

## 2. Case Report

**Figure 1:**
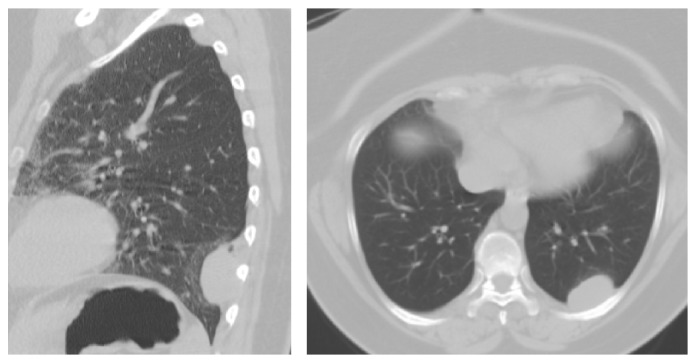
Left sided posterior pleural-based mass.

A 51-year-old woman presented to the emergency department with persistent posterior left sided chest discomfort. Patient had nonspecific symptoms for over one year. Upon admission patient was noted to have a normal EKG findings; X-Ray of the chest was unremarkable. She underwent a CT which revealed a pleural-based mass 3.3 cm × 2.0 cm of the left lower lobe involving the 9th rib posteriorly, in Figure 1. A CT-guided transthoracic core biopsy of the tumor revealed a smooth muscle tumor of the pleura of undetermined malignant potential (SMT-UMP). The specimen showed a bland proliferation of spindle cells with abundant eosinophilic cytoplasm arranged in fascicles. No necrosis and rare mitotic activity were identified. Due to the pattern of spread that has been shown of these tumors (local growth without metastasis) and the lack of high risk features (no necrosis and rare mitotic activity) CT scan of the chest including the upper abdomen was performed for staging purposes and it did not show metastasis.

**Figure 2:**
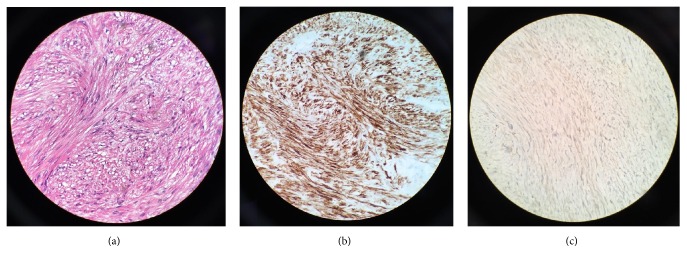
(a) Proliferation of bland spindle cells with elongated nuclei and eosinophilic cytoplasm (hematoxylin and eosin, 400x magnification). (b) Desmin immunohistochemical stain is positive (400x magnification). (c) Beta-catenin is negative (400x magnification).

She was further treated with complete resection of the pleural-based mass and the 9th rib by video-assisted thoracoscopic surgery (VATS). The final pathology examination revealed a well-capsulated SMT-UMP of pleural origin measuring 3.5 × 3.0 × 2.4 cm with no evidence of rib involvement by the tumor, in Figure 2. Patient tolerated the procedure well without any surgical or medical complications. Unfortunately patient was lost to follow-up.  Table 2 shows patient's immunohistochemical staining.

## 3. Discussion

SMT-UMP originating in the pleura are rare. They tend to have a female predominance (12 out of 18 patients) as per current literature review. Patient's age ranges from 21 to 73 years old (mean 42.7). Leiomyosarcomas (LMS) were found mainly in older patients and SMT of UMP on the other hand tend to happen in younger patients. Our case illustrates that SMT-UMP can present in a relatively older age.

SMTs can be found incidentally on imaging studies done for unrelated issues or they can cause symptoms usually related to tumor size (the largest one has been reported as at least 21 cm) and location. In our case a middle aged women presented with nonspecific pleuritic chest pain where fatal conditions need to be ruled out. These patients should undergo a thorough history and physical examination. Radiological studies should be performed with chest X-ray (CXR), computed tomography, and magnetic resonance imaging that helps identify location, size, and radiological structure of the tumor [6].

Pleural tumors tend to grow locally toward the intrathoracic cavity [15]. There is not a single case reported in the literature showing that these tumors did metastasize. Proca et al. [6] reported a case that was followed up without any surgical intervention for four years and it showed that the tumor did grow locally inside and out of the thoracic cavity but no metastasis was reported. However, due to the rarity of these tumors and short follow-ups reported there is not enough data at the moment to determine if these tumors have the ability to metastasize.

Confirmation of diagnosis is always made with tissue sample and histological examination. A CT-guided biopsy can be performed, but it has the potential to seed the tissues directly in its path [6]. If surgical excision of the lesion can be done safely with minimal complications it should be done with diagnostic and treatment purposes instead of diagnostic needle biopsy in order to avoid potential seeding and spread of tumor with malignant potential.

Primary pleural tumors are rare since 75% of pleural tumors represent metastatic disease [13]. The differential diagnosis of spindle cell neoplasms from pleural origin includes smooth muscle tumor, solitary fibrous tumor, metastatic spindle cell carcinoma, synovial sarcoma, fibrosarcoma, malignant peripheral nerve sheath tumor, sarcomatous mesothelioma, and spindle cell thymoma [2].  Table 3 describes the main differences between these entities.

Immunohistochemical staining for smooth muscle actin and desmin provides a definitive diagnosis of smooth muscle origin.

In our patient, microscopic examination showed proliferation of bland spindle cells with elongated nuclei, eosinophilic cytoplasm, and rare mitotic figures. Focal areas of increased cellularity and atypia were present, but no necrosis was identified, in Figure 2. The tumor cells reacted with immunohistochemical stains for desmin, smooth muscle actin, SMMHC, CD34 (focal), and vimentin. Additional immunostains were performed, including S-100, BCL-2, CD99, and beta-catenin. All of these were negative. Less than 2% of the cells showed reactivity with proliferation index Ki-67; see Table 2. The pathological findings were diagnostic of a smooth muscle neoplasm. The absence of pleomorphism, increased mitotic figures, necrosis, and poor differentiation distinguished SMT of UMP from LMS [7].

Even when smooth muscle tumors of the pleura appear benign, well-encapsulated, smooth, and without evidence of necrosis and show rare mitotic activity they can possess malignant potential and present as or transform in LMS; see Table 1 [7].

Primary and preferred treatment is surgical resection if feasible which can be performed using minimally invasive surgery such as VATS, if after surgical resection there are positive margins to consider reresection (preferable option) versus observation (watch and wait approach) of the remaining disease with serial imaging studies during follow-up [6]. Smooth muscle tumors may increase in size with local invasion to the mediastinum and other structures, which can jeopardize complete resection with curative intent. If further surgery is contraindicated and disease was left behind perhaps the role of radiation could be explored [17–20]. Al-Daraji et al. [7] reported a case where there was a concern for possible incomplete resection at the apex and this patient received radiation with the intention to reduce the risk of local recurrence. This patient was reported to be alive at 14 months of follow-up and without recurrence. At present, there is no role for adjuvant chemotherapy.

The prognosis appears to be good if the tumor is excised completely with negative margins, but routine follow-up should not be neglected. Due to the rarity of this entity and relative short follow-ups the behavior of this tumor cannot be properly evaluated.

## 4. Conclusion

Primary SMTs of the pleura are infrequent tumors and should be considered as a differential diagnosis when approaching a pleural mass. It seems to develop from the vascular smooth muscle cells. SMT-UMP tends to affect younger patients and LMS to tends to affect older patients. However, SMT-UMP can present in older patients as in our case. They appear to grow locally and invade nearby structures but there is not yet a single case reporting distant metastasis (the present case was not the exception). Tissue diagnosis and accurate histopathological evaluation are required. Although these tumors seem to possess low malignant potential they can be life threatening (they can grow very large causing serious symptomatology and/or degenerate into malignant tumors) and should be treated as such with appropriate surgical management and close follow-up.

## Figures and Tables

**Table 1:** Clinical and histopathological features of previous case reports and current case report of SMT of the pleura.

Case	Sex	Age	Clinical features	Size^a^ (cm)	Histology	Origin of the tumor	Procedure	Follow-up (months)	Clinical course
1^b^	F	21	Asymptomatic	U	SMT of UMP	Vascular smooth muscle (pleura)	Too large for complete resection	4	Alive at 4 M without enlargement or metastasis
2	M	49	Asymptomatic	18	LMS/IG	No detail	Complete resection	8	Alive at 8 M without recurrence
3	F	23	Asymptomatic	10	SMT of UMP	Vascular smooth muscle (pleura)	Too large for complete resection	6	Alive at 6 M without enlargement or metastasis
4	F	44	Empyema	U	LMS/IG	No detail	Complete resection	2	Alive at 2 M without recurrence
5	F	69	Chest pain	11	LMS/HG	No detail	Complete resection	12	Alive at 12 M without recurrence
6	M	32	Asymptomatic	7 (intrathoracic) + 6 (extrathoracic) = 13	SMT of UMP	No detail (pleura)	Complete resection	12	Alive at 12 M without recurrence
7	M	73	Asymptomatic	At least 21	SMT of UMP→LMS	No detail (pleura)	Possible incomplete resection at the apex and received radiation to reduce the risk of local recurrence	14	Alive at 14 M without recurrence
8	F	55	Asymptomatic	1.5	Leiomyoma	Microvascular wall (pleura)	Complete resection	26	Alive at 26 M without recurrence
9	F	40	Asymptomatic	3.5	SMT of UMP	Microvascular wall (pleura)	Complete resection	17	Alive at 17 M without recurrence
10	M	45	Chest pain	9	Leiomyoma	No detail (pleura)	Complete resection	15	Alive at 15 M without recurrence
11	M	33	Asymptomatic	3	Leiomyoma	No detail	Complete resection	Unknown	Unknown
12	F	50	Chest pain	4	SMT of UMP	Vascular smooth muscle (pleura)	Complete resection	53	Alive at 53 M without recurrence
13	F	48	Chest pain	18	Leiomyoma	No detail (pleura)	Complete resection	18	Alive at 18 M without recurrence
14	F	32	Chest pain	2 tumors, unknown size	Leiomyoma	No detail	Complete resection	57	Recurrence after 1 year during follow-up Underwent to chest wall resection. Alive at 57 M without further recurrence
15	M	43	Chest pain	2	Leiomyoma	No detail	Complete resection	40	Alive at 40 M without recurrence
16	F	28	Chest pain	4.2	Leiomyoma	No detail (intercostal space)	Complete resection	2	Alive at 2 M without recurrence
17	F	33	Chest pain	5.3	Leiomyoma	Vascular smooth muscle (pleura)	Complete resection	14	Alive at 14 M without recurrence
Present case	F	51	Chest pain	3.5	SMT of UMP	No detail	Complete resection	0	—

^a^Maximum diameter of the tumor in cm.

^b^Cases 1–5 from Moran et al. [5]; case 6 from Proca et al. [6]; case 7 from Al-Daraji et al. [7]; case 8 from Nose et al. [8]; case 9 from Tanaka et al. [9]; case 10 from Qiu et al. [10]; case 11 from Mochizuki et al. [11]; case 12 from Turhan et al. [12]; case 13 from Rodríguez et al. [13]; cases 14 and 15 from Kuman et al. [14]; case 16 from Nakada et al. [15]; case 17 from Ziyade et al. [16].

Note: U, unresectable tumors that were only debulked at surgery; SMT, smooth muscle tumor; UMP, undetermined malignant potential; LMS, leiomyosarcoma; IG, intermediate grade; HG, high grade.

**Table 2:** Immunohistochemical staining.

Immunohistochemical staining	Result
CD34 (focal)	Positive
Smooth muscle actin	Positive
SMMHC	Positive
Desmin	Positive
Vimentin	Positive
CD99	Negative
Beta-catenin	Negative
S-100	Negative
BCL-2	Negative
Ki-67	Less than 2% cells showing nuclear staining
Cytokeratin AE1/AE3	Negative

**Table 3:** Immunohistochemical pattern in pleural spindle cell neoplasms [6].

Tumor	Vimentin	SMA	HHF-35	SMMHC	Desmin	CD34	S100	BCL-2	CD99	Cytokeratin
Smooth muscle tumor	+	+	+	+	+	+/−	−	−	−	−
Solitary fibrous tumor	+	−/+	−/+	−/+	−	+	−	+	+	−
Metastatic spindle cell carcinoma	+/−	−	−	−	−	−	−	−	−	+
Synovial sarcoma	+	−	−	−	−	−	−	+	+/−	+/−
Fibrosarcoma	+	−	−	−	−	−	−	−/+	−	−
Malignant peripheral nerve sheath tumor	+	−	−	−	−	−	−/+	−/+	−	−
Sarcomatous mesothelioma	+	−	−	−	−/+	−	−/+	−/+	−/+	+
Spindle cell thymoma	−	−	−	−	−	−	−	−/+	−	+

SMA: smooth muscle actin; HHF-35: actin muscle specific; SMMHC, smooth muscle myosin-heavy chain; +, positive staining; +/−, usually positive; −/+, rarely positive; and −, negative.
